# (4R*,4aS*,4bS*,5R*,10aR*)-4-Hy­droxy-4a,5-dimethyl-2-(propan-2-yl)-1,4,4a,4b,5,6,7,8,10,10a-deca­hydro­phenan­thren-1-one

**DOI:** 10.1107/S1600536811048008

**Published:** 2011-11-16

**Authors:** Ignez Caracelli, Julio Zukerman-Schpector, André T. Lousada Machado, Timothy J. Brocksom, M. Lúcia Ferreira, Edward R. T. Tiekink

**Affiliations:** a BioMat-Departamento de Física, Universidade Federal de São Carlos, CP 676, 13565-905, São Carlos, SP, Brazil; b Laboratório de Cristalografia, Estereodinâmica e Modelagem Molecular, Departamento de Química, Universidade Federal de São Carlos, CP 676, 13565-905, São Carlos, SP, Brazil; c Departamento de Química, Universidade Federal de São Carlos, 13565-905 São Carlos, SP, Brazil; d Department of Chemistry, University of Malaya, 50603 Kuala Lumpur, Malaysia

## Abstract

In the title compound, C_19_H_28_O_2_, the A ring adopts a chair conformation. Both the *B* and *C* rings adopt envelope conformations with the C atoms common to both rings and adjacent to the carbonyl and hydroxyl groups, respectively, lying 0.604 (3) and 0.634 (3) Å out of the mean planes defined by the remaining five C atoms of rings *B* and *C*, respectively (r.m.s. deviations = 0.0100 and 0.0157 Å, respectively). The formation of linear supra­molecular *C*(7) chains along the *a* axis mediated by hy­droxy-O—H⋯O(carbon­yl) hydrogen bonds is the most prominent feature of the crystal packing.

## Related literature

For background to the biological activity of some diterpene compounds, see: Guo *et al.* (2011 ▶); Slusarczyk *et al.* (2011 ▶). For the synthesis, see: Ferreira (2002 ▶). For conformational analysis, see: Cremer & Pople (1975 ▶).
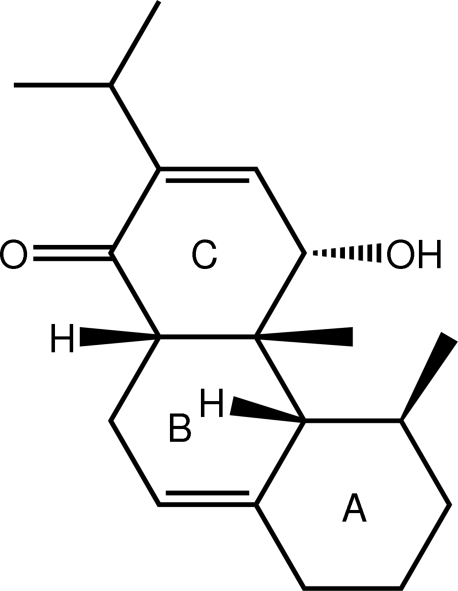


## Experimental

### 

#### Crystal data

C_19_H_28_O_2_
*M* *_r_* = 288.41
Orthorhombic, 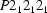

*a* = 6.5507 (9) Å
*b* = 11.733 (1) Å
*c* = 22.338 (3) Å
*V* = 1716.9 (4) Å^3^
*Z* = 4
Mo *K*α radiation
μ = 0.07 mm^−1^
*T* = 290 K
0.15 × 0.12 × 0.09 mm

#### Data collection

Enraf–Nonius CAD-4 Mach 3 diffractometer
2272 measured reflections
1945 independent reflections
1077 reflections with *I* > 2σ(*I*)
*R*_int_ = 0.038
3 standard reflections every 30 min  intensity decay: 2.0%

#### Refinement

*R*[*F* ^2^ > 2σ(*F* ^2^)] = 0.037
*wR*(*F* ^2^) = 0.122
*S* = 1.02
1945 reflections
191 parameters
H-atom parameters constrained
Δρ_max_ = 0.15 e Å^−3^
Δρ_min_ = −0.11 e Å^−3^

### 

Data collection: *CAD-4 Software* (Enraf–Nonius, 1989 ▶); cell refinement: *CAD-4 Software*; data reduction: *MolEN* (Fair, 1990 ▶); program(s) used to solve structure: *SIR92* (Altomare *et al.*, 1999 ▶); program(s) used to refine structure: *SHELXL97* (Sheldrick, 2008 ▶); molecular graphics: *ORTEP-3* (Farrugia, 1997 ▶), *DIAMOND* (Brandenburg, 2006 ▶) and *MarvinSketch* (Chemaxon, 2009 ▶); software used to prepare material for publication: *publCIF* (Westrip, 2010 ▶).

## Supplementary Material

Crystal structure: contains datablock(s) global, I. DOI: 10.1107/S1600536811048008/hb6477sup1.cif

Structure factors: contains datablock(s) I. DOI: 10.1107/S1600536811048008/hb6477Isup2.hkl

Additional supplementary materials:  crystallographic information; 3D view; checkCIF report

## Figures and Tables

**Table 1:** Hydrogen-bond geometry (Å, °)

*D*—H⋯*A*	*D*—H	H⋯*A*	*D*⋯*A*	*D*—H⋯*A*
O2—H2*o*⋯O1^i^	0.82	2.02	2.804 (3)	160

Symmetry code: (i) 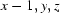.

**Fig. 1.**
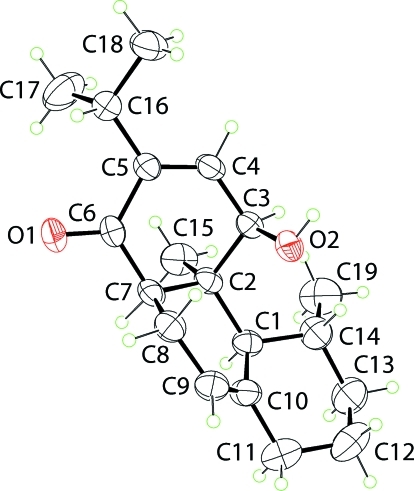
The molecular structure of compound (I) showing displacement ellipsoids at the 30% probability level (arbitrary spheres for the H atoms).

**Fig. 2.**
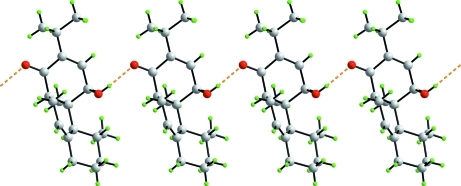
A view of the linear supramolecular chain along the a axis in (I). The hydroxy-O—H···O(carbonyl) hydrogen bonds are represented by orange dashed lines.

**Fig. 3.**
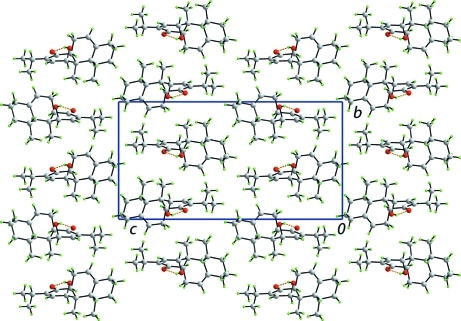
A view in projection down the a axis of the unit-cell contents of (I). The hydroxy-O—H···O(carbonyl) hydrogen bonds are represented by orange dashed lines.

## supplementary crystallographic information

### Comment

Natural diterpenes exhibit a wide range of biological activities such as neuroprotectives (Guo *et al.*, 2011) and as anti-plasmodials and anti-trypanocidals (Slusarczyk *et al.*, 2011). While aiming at the synthesis of some hydrophenanthrene diterpenes, a series of new intermediates were obtained and among them was the title compound (Ferreira, 2002), (I), which has been characterized crystallographically.

The A ring in (I), Fig. 1, has a chair conformation. Each of the B and C rings presents a half-chair conformation with atom C7 lying 0.604 (3) Å and C2 lying 0.634 (3) Å out of the approximate plane defined by the remaining five C atoms of rings B and C, respectively (r.m.s. deviation 0.0100 and 0.0157 Å for rings B and C, respectively). The ring puckering parameters are: q_2_ = 0.040 (4), 0.348 (3), 0.367 (3) Å; q_3_ = 0.530 (4), 0.265 (3), 0.269 (3) Å; QT = 0.531 (4), 0.438 (3), 0.455 (3) Å; and θ = 3.9 (4), 52.7 (4), 53.7 (4)°, for rings A, B and C, respectively (Cremer & Pople, 1975).

In the crystal packing, the molecules are linked through O–H···O hydrogen bonds to form linear supramolecular chains along the *a* axis, Fig. 2 and Table 1. Chains pack in the crystal structure with no specific intermolecular interactions operating between them, Fig. 3.

### Experimental

The detailed synthesis of the title compound is described in a Ph.D. thesis (Ferreira, 2002). Crystals were grown by slow evaporation from its hexane solution held at 293 K. ^1^H-NMR (CDCl_3_, 400 MHz): δ (p.p.m.): 6.47 (d, 1H, J = 5.4 Hz); 5.50 (d, 1H, J = 4.5 Hz); 4.4 (d, 1H, J = 5.4 Hz); 2.87 (heptet, 1H, J = 6.8 Hz); 2.39 (d, 1H, J = 3.7 Hz); 2.29–2.33 (m, 1H); 2.08–2.13 (m, 2H); 1.98–2.03 (m, 2H); 1.68–1.78 (m, 1H); 1.68–1.78 (m, 2H); 1.35 (dt, 2H, J_1_ = 12.8 and J_2_ = 3.6 Hz); 1.21 (d, 3H, J = 6.5 Hz); 1.16 (s, 3H); 1.06 (d, 3H, J = 6.9 Hz); 1.02 (d, 3H, J = 6.9 Hz); δ(OH) not obs. ^13^C (CDCl_3_, 100 MHz) δ (p.p.m.): 202.5; 142.2; 141.8; 136.0; 118.3; 71.0; 53.9; 53.1; 37.6; 36.7; 36.7; 33.6; 29.0; 27.5; 26.1; 26.0; 25.2; 21.4; 21.4 Analysis found: C 78.98, H 9.79%. C_19_H_28_O_2_ requires: C 79.12, H 9.79%.

### Refinement

The H atoms were geometrically placed (C—H = 0.93–0.98 Å; O—H = 0.82 Å) and refined as riding with *U_iso_*(H) = 1.2*U_eq_*(C) and *U_iso_*(H) = 1.5*U_eq_*(methyl-C,*O*). The absolute structure was based on that of a starting material used in the synthesis (Ferreira, 2002). In the absence of significant anamolous dispersion effects, 287 Friedel pairs were merged in the final refinement cycles.

### Crystal data

**Table:**

C_19_H_28_O_2_	*F*(000) = 632
*M**_r_* = 288.41	*D*_x_ = 1.116 Mg m^−^^3^
Orthorhombic, *P*2_1_2_1_2_1_	Mo *K*α radiation, λ = 0.71073 Å
Hall symbol: P 2ac 2ab	Cell parameters from 23 reflections
*a* = 6.5507 (9) Å	θ = 9.1–16.5°
*b* = 11.733 (1) Å	µ = 0.07 mm^−^^1^
*c* = 22.338 (3) Å	*T* = 290 K
*V* = 1716.9 (4) Å^3^	Irregular, colourless
*Z* = 4	0.15 × 0.12 × 0.09 mm

### Data collection

**Table:**

Enraf–Nonius CAD-4 Mach 3 diffractometer	*R*_int_ = 0.038
Radiation source: fine-focus sealed tube	θ_max_ = 26.0°, θ_min_ = 2.0°
graphite	*h* = −1→8
ω/–2θ scans	*k* = −14→0
2272 measured reflections	*l* = −27→0
1945 independent reflections	3 standard reflections every 30 min
1077 reflections with *I* > 2σ(*I*)	intensity decay: 2.0%

### Refinement

**Table:**

Refinement on *F*^2^	Primary atom site location: structure-invariant direct methods
Least-squares matrix: full	Secondary atom site location: difference Fourier map
*R*[*F*^2^ > 2σ(*F*^2^)] = 0.037	Hydrogen site location: inferred from neighbouring sites
*wR*(*F*^2^) = 0.122	H-atom parameters constrained
*S* = 1.02	*w* = 1/[σ^2^(*F*_o_^2^) + (0.0557*P*)^2^ + 0.0441*P*] where *P* = (*F*_o_^2^ + 2*F*_c_^2^)/3
1945 reflections	(Δ/σ)_max_ < 0.001
191 parameters	Δρ_max_ = 0.15 e Å^−^^3^
0 restraints	Δρ_min_ = −0.11 e Å^−^^3^

### Special details

**Table:**

Geometry. All s.u.'s (except the s.u. in the dihedral angle between two l.s. planes) are estimated using the full covariance matrix. The cell s.u.'s are taken into account individually in the estimation of s.u.'s in distances, angles and torsion angles; correlations between s.u.'s in cell parameters are only used when they are defined by crystal symmetry. An approximate (isotropic) treatment of cell s.u.'s is used for estimating s.u.'s involving l.s. planes.
Refinement. Refinement of *F*^2^ against ALL reflections. The weighted *R*-factor *wR* and goodness of fit *S* are based on *F*^2^, conventional *R*-factors *R* are based on *F*, with *F* set to zero for negative *F*^2^. The threshold expression of *F*^2^ > 2σ(*F*^2^) is used only for calculating *R*-factors(gt) *etc*. and is not relevant to the choice of reflections for refinement. *R*-factors based on *F*^2^ are statistically about twice as large as those based on *F*, and *R*- factors based on ALL data will be even larger.

### Fractional atomic coordinates and isotropic or equivalent isotropic displacement parameters (Å^2^)

**Table:**

	*x*	*y*	*z*	*U*_iso_*/*U*_eq_	
C1	0.5700 (5)	0.1474 (3)	0.87731 (12)	0.0536 (8)	
H1	0.6626	0.1974	0.8996	0.064*	
C2	0.6209 (4)	0.1683 (2)	0.80946 (13)	0.0488 (8)	
C3	0.4355 (4)	0.1501 (3)	0.76804 (12)	0.0478 (7)	
H3	0.3371	0.2113	0.7758	0.057*	
C4	0.4904 (5)	0.1535 (3)	0.70352 (13)	0.0527 (8)	
H4	0.3867	0.1723	0.6769	0.063*	
C5	0.6731 (5)	0.1322 (3)	0.68010 (13)	0.0508 (7)	
C6	0.8399 (5)	0.1005 (3)	0.72195 (15)	0.0559 (8)	
C7	0.7936 (4)	0.0895 (2)	0.78799 (14)	0.0526 (8)	
H7	0.9173	0.1121	0.8096	0.063*	
C8	0.7588 (5)	−0.0363 (3)	0.80105 (14)	0.0616 (9)	
H8A	0.6508	−0.0653	0.7755	0.074*	
H8B	0.8823	−0.0789	0.7925	0.074*	
C9	0.7016 (6)	−0.0514 (3)	0.86456 (15)	0.0656 (9)	
H9	0.7258	−0.1223	0.8818	0.079*	
C10	0.6191 (5)	0.0279 (3)	0.89854 (14)	0.0592 (8)	
C11	0.5787 (7)	0.0058 (4)	0.96343 (16)	0.0875 (13)	
H11A	0.6721	0.0509	0.9874	0.105*	
H11B	0.6046	−0.0739	0.9720	0.105*	
C12	0.3627 (8)	0.0344 (4)	0.98085 (18)	0.1012 (15)	
H12A	0.2688	−0.0165	0.9606	0.121*	
H12B	0.3454	0.0245	1.0237	0.121*	
C13	0.3167 (8)	0.1551 (3)	0.96402 (16)	0.0900 (12)	
H13A	0.4014	0.2053	0.9880	0.108*	
H13B	0.1753	0.1712	0.9738	0.108*	
C14	0.3521 (6)	0.1821 (3)	0.89755 (16)	0.0699 (10)	
H14	0.2546	0.1368	0.8743	0.084*	
C15	0.6977 (6)	0.2914 (3)	0.80117 (15)	0.0750 (11)	
H15A	0.5933	0.3438	0.8135	0.113*	
H15B	0.7300	0.3041	0.7598	0.113*	
H15C	0.8177	0.3030	0.8251	0.113*	
C16	0.7253 (5)	0.1371 (3)	0.61470 (13)	0.0627 (9)	
H16	0.8109	0.0708	0.6056	0.075*	
C17	0.8497 (8)	0.2431 (4)	0.60174 (17)	0.1106 (16)	
H17A	0.8863	0.2445	0.5601	0.166*	
H17B	0.9713	0.2425	0.6257	0.166*	
H17C	0.7704	0.3095	0.6111	0.166*	
C18	0.5396 (6)	0.1315 (4)	0.57472 (15)	0.0879 (13)	
H18A	0.5818	0.1327	0.5336	0.132*	
H18B	0.4530	0.1958	0.5826	0.132*	
H18C	0.4659	0.0624	0.5826	0.132*	
C19	0.2980 (9)	0.3082 (3)	0.88848 (17)	0.1022 (16)	
H19A	0.1605	0.3215	0.9015	0.153*	
H19B	0.3100	0.3272	0.8468	0.153*	
H19C	0.3898	0.3547	0.9114	0.153*	
O1	1.0090 (3)	0.0749 (2)	0.70292 (10)	0.0824 (8)	
O2	0.3390 (3)	0.04403 (17)	0.78121 (9)	0.0563 (6)	
H2o	0.2390	0.0359	0.7595	0.085*	

### Atomic displacement parameters (Å^2^)

**Table:**

	*U*^11^	*U*^22^	*U*^33^	*U*^12^	*U*^13^	*U*^23^
C1	0.0577 (17)	0.0556 (18)	0.0474 (17)	−0.0035 (17)	−0.0097 (15)	−0.0039 (15)
C2	0.0480 (18)	0.0450 (17)	0.0533 (17)	−0.0047 (15)	−0.0104 (15)	0.0007 (13)
C3	0.0434 (16)	0.0470 (17)	0.0529 (17)	0.0061 (15)	−0.0055 (13)	0.0023 (14)
C4	0.0456 (16)	0.0577 (19)	0.0546 (18)	0.0020 (16)	−0.0140 (15)	0.0068 (15)
C5	0.0432 (16)	0.0584 (19)	0.0509 (16)	−0.0044 (16)	−0.0060 (15)	0.0036 (15)
C6	0.0357 (15)	0.068 (2)	0.064 (2)	−0.0068 (16)	−0.0025 (16)	0.0015 (16)
C7	0.0381 (16)	0.0631 (19)	0.0566 (19)	−0.0061 (15)	−0.0127 (15)	0.0056 (16)
C8	0.0565 (18)	0.060 (2)	0.068 (2)	0.0132 (17)	−0.0010 (17)	0.0056 (17)
C9	0.069 (2)	0.057 (2)	0.071 (2)	0.0115 (19)	−0.0020 (18)	0.0172 (17)
C10	0.0614 (19)	0.062 (2)	0.0543 (19)	0.0002 (18)	−0.0080 (17)	0.0110 (17)
C11	0.112 (3)	0.089 (3)	0.062 (2)	−0.001 (3)	0.001 (2)	0.012 (2)
C12	0.127 (4)	0.109 (3)	0.068 (2)	−0.018 (3)	0.028 (3)	0.005 (2)
C13	0.094 (3)	0.108 (3)	0.068 (2)	0.009 (3)	0.014 (2)	−0.017 (2)
C14	0.072 (2)	0.074 (2)	0.064 (2)	0.007 (2)	0.002 (2)	−0.0152 (17)
C15	0.092 (3)	0.054 (2)	0.079 (2)	−0.018 (2)	−0.014 (2)	0.0031 (17)
C16	0.0524 (18)	0.082 (2)	0.0536 (19)	−0.0020 (19)	−0.0015 (16)	0.0041 (17)
C17	0.129 (4)	0.126 (4)	0.077 (3)	−0.042 (4)	0.018 (3)	0.021 (3)
C18	0.077 (2)	0.130 (3)	0.057 (2)	0.019 (3)	−0.017 (2)	−0.009 (2)
C19	0.129 (4)	0.091 (3)	0.087 (3)	0.044 (3)	0.007 (3)	−0.018 (2)
O1	0.0360 (11)	0.143 (2)	0.0685 (15)	0.0035 (15)	0.0004 (12)	0.0002 (15)
O2	0.0438 (11)	0.0629 (13)	0.0623 (13)	−0.0109 (12)	−0.0077 (11)	−0.0014 (11)

### Geometric parameters (Å, °)

**Table:**

C1—C10	1.514 (4)	C11—H11B	0.9700
C1—C14	1.552 (5)	C12—C13	1.495 (6)
C1—C2	1.571 (4)	C12—H12A	0.9700
C1—H1	0.9800	C12—H12B	0.9700
C2—C7	1.538 (4)	C13—C14	1.536 (5)
C2—C15	1.541 (4)	C13—H13A	0.9700
C2—C3	1.542 (4)	C13—H13B	0.9700
C3—O2	1.427 (3)	C14—C19	1.535 (5)
C3—C4	1.486 (4)	C14—H14	0.9800
C3—H3	0.9800	C15—H15A	0.9600
C4—C5	1.330 (4)	C15—H15B	0.9600
C4—H4	0.9300	C15—H15C	0.9600
C5—C6	1.485 (4)	C16—C18	1.511 (5)
C5—C16	1.501 (4)	C16—C17	1.515 (5)
C6—O1	1.224 (4)	C16—H16	0.9800
C6—C7	1.512 (5)	C17—H17A	0.9600
C7—C8	1.522 (4)	C17—H17B	0.9600
C7—H7	0.9800	C17—H17C	0.9600
C8—C9	1.478 (4)	C18—H18A	0.9600
C8—H8A	0.9700	C18—H18B	0.9600
C8—H8B	0.9700	C18—H18C	0.9600
C9—C10	1.317 (4)	C19—H19A	0.9600
C9—H9	0.9300	C19—H19B	0.9600
C10—C11	1.496 (5)	C19—H19C	0.9600
C11—C12	1.505 (6)	O2—H2o	0.8200
C11—H11A	0.9700		
			
C10—C1—C14	110.3 (3)	H11A—C11—H11B	107.9
C10—C1—C2	113.7 (2)	C13—C12—C11	109.6 (4)
C14—C1—C2	115.8 (3)	C13—C12—H12A	109.7
C10—C1—H1	105.3	C11—C12—H12A	109.7
C14—C1—H1	105.3	C13—C12—H12B	109.7
C2—C1—H1	105.3	C11—C12—H12B	109.7
C7—C2—C15	106.6 (3)	H12A—C12—H12B	108.2
C7—C2—C3	108.0 (2)	C12—C13—C14	114.1 (3)
C15—C2—C3	108.3 (2)	C12—C13—H13A	108.7
C7—C2—C1	111.3 (2)	C14—C13—H13A	108.7
C15—C2—C1	109.4 (2)	C12—C13—H13B	108.7
C3—C2—C1	113.0 (2)	C14—C13—H13B	108.7
O2—C3—C4	109.3 (2)	H13A—C13—H13B	107.6
O2—C3—C2	110.2 (2)	C19—C14—C13	106.9 (3)
C4—C3—C2	112.8 (2)	C19—C14—C1	115.2 (4)
O2—C3—H3	108.1	C13—C14—C1	111.5 (3)
C4—C3—H3	108.1	C19—C14—H14	107.6
C2—C3—H3	108.1	C13—C14—H14	107.6
C5—C4—C3	126.5 (3)	C1—C14—H14	107.6
C5—C4—H4	116.8	C2—C15—H15A	109.5
C3—C4—H4	116.8	C2—C15—H15B	109.5
C4—C5—C6	117.5 (3)	H15A—C15—H15B	109.5
C4—C5—C16	125.5 (3)	C2—C15—H15C	109.5
C6—C5—C16	117.0 (3)	H15A—C15—H15C	109.5
O1—C6—C5	120.6 (3)	H15B—C15—H15C	109.5
O1—C6—C7	120.0 (3)	C5—C16—C18	113.0 (3)
C5—C6—C7	119.2 (3)	C5—C16—C17	109.8 (3)
C6—C7—C8	107.5 (3)	C18—C16—C17	110.9 (3)
C6—C7—C2	113.6 (2)	C5—C16—H16	107.6
C8—C7—C2	114.4 (3)	C18—C16—H16	107.6
C6—C7—H7	107.0	C17—C16—H16	107.6
C8—C7—H7	107.0	C16—C17—H17A	109.5
C2—C7—H7	107.0	C16—C17—H17B	109.5
C9—C8—C7	109.8 (3)	H17A—C17—H17B	109.5
C9—C8—H8A	109.7	C16—C17—H17C	109.5
C7—C8—H8A	109.7	H17A—C17—H17C	109.5
C9—C8—H8B	109.7	H17B—C17—H17C	109.5
C7—C8—H8B	109.7	C16—C18—H18A	109.5
H8A—C8—H8B	108.2	C16—C18—H18B	109.5
C10—C9—C8	124.9 (3)	H18A—C18—H18B	109.5
C10—C9—H9	117.6	C16—C18—H18C	109.5
C8—C9—H9	117.6	H18A—C18—H18C	109.5
C9—C10—C11	120.5 (3)	H18B—C18—H18C	109.5
C9—C10—C1	124.1 (3)	C14—C19—H19A	109.5
C11—C10—C1	115.2 (3)	C14—C19—H19B	109.5
C10—C11—C12	112.2 (4)	H19A—C19—H19B	109.5
C10—C11—H11A	109.2	C14—C19—H19C	109.5
C12—C11—H11A	109.2	H19A—C19—H19C	109.5
C10—C11—H11B	109.2	H19B—C19—H19C	109.5
C12—C11—H11B	109.2	C3—O2—H2o	109.5
			
C10—C1—C2—C7	−26.9 (3)	C15—C2—C7—C8	171.6 (3)
C14—C1—C2—C7	−156.1 (3)	C3—C2—C7—C8	−72.2 (3)
C10—C1—C2—C15	−144.5 (3)	C1—C2—C7—C8	52.4 (3)
C14—C1—C2—C15	86.3 (4)	C6—C7—C8—C9	−176.9 (3)
C10—C1—C2—C3	94.8 (3)	C2—C7—C8—C9	−49.8 (4)
C14—C1—C2—C3	−34.5 (4)	C7—C8—C9—C10	23.6 (5)
C7—C2—C3—O2	74.3 (3)	C8—C9—C10—C11	−176.3 (3)
C15—C2—C3—O2	−170.6 (3)	C8—C9—C10—C1	0.0 (6)
C1—C2—C3—O2	−49.2 (3)	C14—C1—C10—C9	133.7 (4)
C7—C2—C3—C4	−48.2 (3)	C2—C1—C10—C9	1.7 (5)
C15—C2—C3—C4	67.0 (3)	C14—C1—C10—C11	−49.8 (4)
C1—C2—C3—C4	−171.7 (3)	C2—C1—C10—C11	178.2 (3)
O2—C3—C4—C5	−99.1 (4)	C9—C10—C11—C12	−128.9 (4)
C2—C3—C4—C5	23.9 (4)	C1—C10—C11—C12	54.5 (5)
C3—C4—C5—C6	0.8 (5)	C10—C11—C12—C13	−55.1 (5)
C3—C4—C5—C16	−179.5 (3)	C11—C12—C13—C14	55.9 (5)
C4—C5—C6—O1	176.7 (3)	C12—C13—C14—C19	−179.9 (4)
C16—C5—C6—O1	−3.1 (5)	C12—C13—C14—C1	−53.1 (5)
C4—C5—C6—C7	2.3 (4)	C10—C1—C14—C19	169.7 (3)
C16—C5—C6—C7	−177.4 (3)	C2—C1—C14—C19	−59.4 (4)
O1—C6—C7—C8	−77.0 (4)	C10—C1—C14—C13	47.6 (4)
C5—C6—C7—C8	97.4 (3)	C2—C1—C14—C13	178.5 (3)
O1—C6—C7—C2	155.4 (3)	C4—C5—C16—C18	−18.6 (5)
C5—C6—C7—C2	−30.2 (4)	C6—C5—C16—C18	161.2 (3)
C15—C2—C7—C6	−64.5 (3)	C4—C5—C16—C17	105.8 (4)
C3—C2—C7—C6	51.7 (3)	C6—C5—C16—C17	−74.5 (4)
C1—C2—C7—C6	176.3 (2)		

### Hydrogen-bond geometry (Å, °)

**Table:**

*D*—H···*A*	*D*—H	H···*A*	*D*···*A*	*D*—H···*A*
O2—H2o···O1^i^	0.82	2.02	2.804 (3)	160

Symmetry codes: (i) *x*−1, *y*, *z*.
